# The Role and Therapeutic Potential of Macropinocytosis in Cancer

**DOI:** 10.3389/fphar.2022.919819

**Published:** 2022-08-15

**Authors:** Zejing Qiu, Wencheng Liu, Qianru Zhu, Kun Ke, Qicong Zhu, Weiwei Jin, Shuxian Yu, Zuyi Yang, Lin Li, Xiaochen Sun, Shuyi Ren, Yanfen Liu, Zhiyu Zhu, Jiangping Zeng, Xiaoyu Huang, Yan Huang, Lu Wei, Mengmeng Ma, Jun Lu, Xiaoyang Chen, Yiping Mou, Tian Xie, Xinbing Sui

**Affiliations:** ^1^ Department of Medical Oncology and School of Pharmacy, The Affiliated Hospital of Hangzhou Normal University, Hangzhou Normal University, Hangzhou, China; ^2^ Key Laboratory of Elemene Class Anti-Cancer Chinese Medicines, Engineering Laboratory of Development and Application of Traditional Chinese Medicines, Collaborative Innovation Center of Traditional Chinese Medicines of Zhejiang Province, Hangzhou Normal University, Hangzhou, China; ^3^ Department of Gastrointestinal-Pancreatic Surgery, General Surgery, Zhejiang Provincial People’s Hospital, People’s Hospital of Hangzhou Medical College, Hangzhou, China

**Keywords:** macropinocytosis, promoting cancer growth, tumor microenvironment, methuosis, anticancer therapies

## Abstract

Macropinocytosis, a unique endocytosis pathway characterized by nonspecific internalization, has a vital role in the uptake of extracellular substances and antigen presentation. It is known to have dual effects on cancer cells, depending on cancer type and certain microenvironmental conditions. It helps cancer cells survive in nutrient-deficient environments, enhances resistance to anticancer drugs, and promotes invasion and metastasis. Conversely, overexpression of the RAS gene alongside drug treatment can lead to methuosis, a novel mode of cell death. The survival and proliferation of cancer cells is closely related to macropinocytosis in the tumor microenvironment (TME), but identifying how these cells interface with the TME is crucial for creating drugs that can limit cancer progression and metastasis. Substantial progress has been made in recent years on designing anticancer therapies that utilize the effects of macropinocytosis. Both the induction and inhibition of macropinocytosis are useful strategies for combating cancer cells. This article systematically reviews the general mechanisms of macropinocytosis, its specific functions in tumor cells, its occurrence in nontumor cells in the TME, and its application in tumor therapies. The aim is to elucidate the role and therapeutic potential of macropinocytosis in cancer treatment.

## Introduction

The development of new anticancer strategies is aided by understanding the molecular mechanisms that afford growth and survival advantages to cancer cells (Zhao et al., 2020). One such adaptive strategy of cancer cells to meet their metabolic needs is the ability to develop alternative nutrient access pathways (Recouvreux and Commisso, 2017). For example, macropinocytosis can enhance the survival, metastasis, and therapeutic resistance of cancer cells (Jayashankar and Edinger, 2020). This ability is essential to cancer cells, and anticancer strategies can be designed around the targeting of macropinocytosis. Macropinocytosis-mediated tumor growth and drug resistance have recently become hot topics in cancer research. This review describes the macropinocytotic mechanisms that affect cancerous growth, resistance, and metastasis. Most significantly, the abnormal function of macropinocytosis is shown to induce methuosis, a new model of cell death. Secondly, the impacts of microenvironmental components on tumor cell growth *via* macropinocytosis are described. Thirdly, anticancer strategies targeting macropinocytosis are reviewed. These include the delivery of chemotherapeutic drugs *via* macropinocytosis to improve their efficacy, the utilization of macropinocytosis inhibitors, and the induction of methuosis. Finally, future research directions are discussed.

## Definition and Origin of Macropinocytosis

Macropinocytosis is clathrin- and caveolin-independent endocytosis (Puccini et al., 2022). This morphological phenomenon—the internalization of extracellular fluids by cytoplasmic membrane folding (King and Kay, 2019)—was first described in the 1930s. It can occur spontaneously or be activated by growth factors, chemokines, or Toll-like receptors, although both cases require the presence of critical signaling hubs. Macropinocytosis signaling pathways form robust submembranous actin networks that induce membrane ruffling and ultimately lead to macropinosome closure (Marques et al., 2017). Macropinocytosis facilitates the large-scale, nonselective internalization of solute molecules in various cell types, including immune cells, fibroblasts, and cancer cells (Hetzenecker et al., 2016; Lin et al., 2020). It enables immune cells to absorb a vast number of pathogens, facilitating immune surveillance and response shaping (Canton, 2018). In solid tumors, macropinocytosis is also a metabolic adaptation in response to nutrient starvation and metabolic stresses. For example, amino acids such as glutamine are essential for cell growth. In RAS-mutant cancers, macropinocytosis enables cancer cells to absorb extracellular proteins which they degrade to amino acids to fuel cell survival and proliferation (Commisso et al., 2013).

**FIGURE 1:**
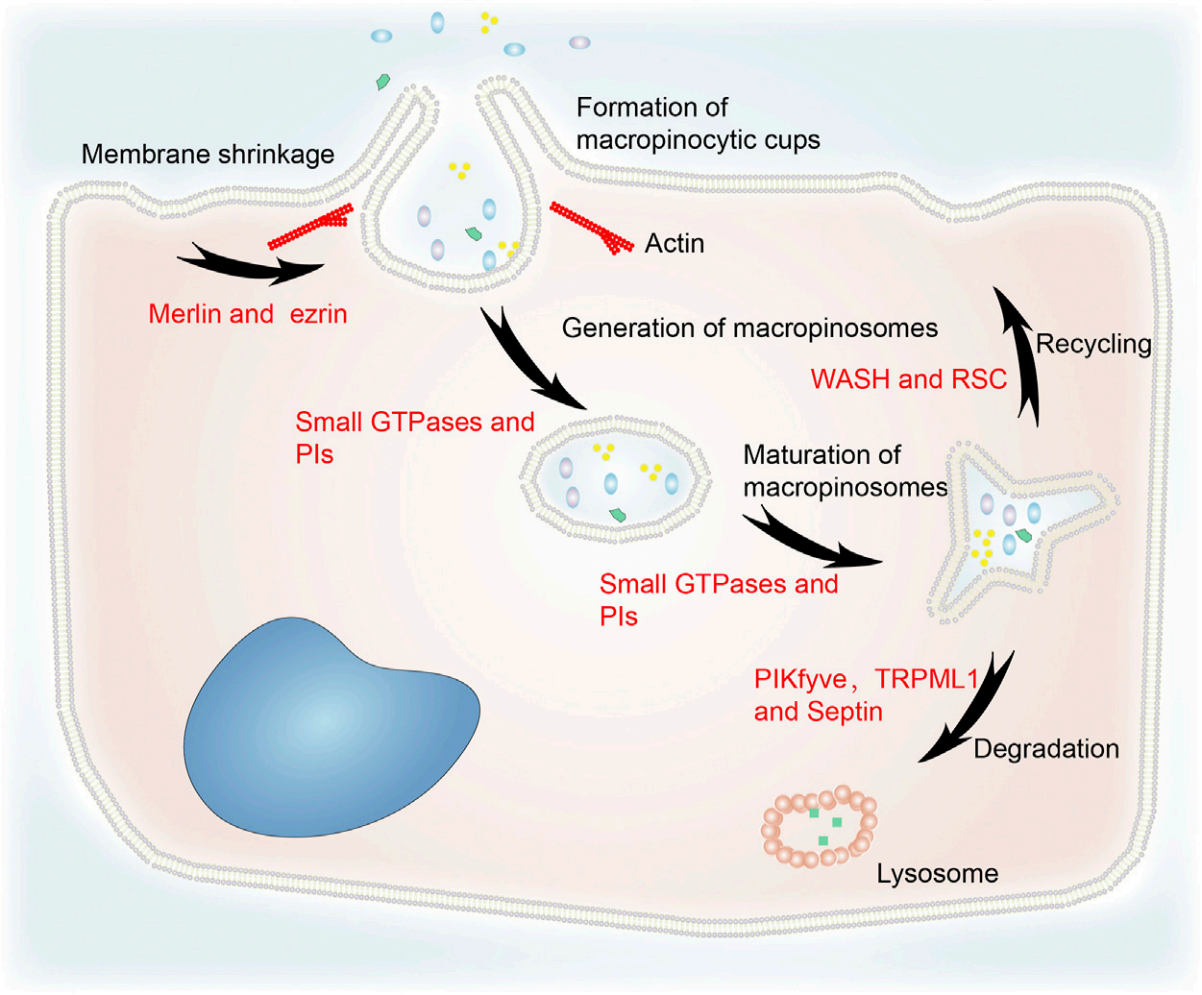
The general process of macropinocytosis including cytoplasmic membrane shrinkage, formation of macropinocytic cups, generation and maturation of macropinosomes, fusion of macropinosomes and lysosomes, recycling, and degradation. Merlin and ezrin are involved in cytoskeleton remodeling. Small GTPases and PIs are essential for the generation and maturation of macropinosomes. WASH and RSC regulate recycling to the cytoplasmic membrane. PIKfyve, TRPML1 and septin are implicated in the degradation of macropinosomes *via* their fusion with lysosomes.

Although macropinocytosis varies between different cell types, the same molecular machinery is utilized. The main steps include cytoplasmic membrane shrinkage, formation of macropinocytic cups, generation and maturation of macropinosomes, macropinosome–lysosome fusion, recycling, and degradation (Figure 1) (Song et al., 2020). Macropinocytosis is initiated by cup-shaped extensions of the plasma membrane, often known as circular ruffles, that are driven by actin polymerization (Ha et al., 2016; Palm, 2019). The formation of macropinosomes thus involves cytoskeleton remodeling. Merlin deficiency was shown to prime the membrane:cytoskeleton interface for epidermal growth factor (EGF)-induced macropinocytosis by increasing cortical ezrin (Chiasson-MacKenzie et al., 2018). The activation of small GTPases was implicated in macropinocytosis by an insertional mutant (Williams et al., 2019). Phosphoinositides (PIs), which transport specific proteins to the endocytic vesicles at a specific time, are also essential for macropinocytosis (Schink et al., 2021).

Loss of liquid or membrane make vesicles more concentrated. Increasing osmotic pressure can cause macropinosomes to lose more surface area than bulk during fission (Hamilton et al., 2007). Therefore, the concentration stage is likely to be fundamental to the maturation of macropinosomes. In addition, small GTPases and PIs regulate macropinosome maturation (Egami et al., 2014).

After maturation, macropinosomes undergo circulation and digestion processes. Their rapid volume loss is mediated by a two-pore channel that transports sodium ions (Freeman et al., 2020). This process reduces the hydrostatic pressure in the macropinosome and facilitates the recycling pathways that return the endocytosed lipids and proteins to the plasma membrane (King and Smythe, 2020). The WASH (WASP and SCAR homologs) complex and retromer sorting complex (RSC) interact with the retromer complex at both early and late phases of macropinosome maturation to mediate recycling (Buckley et al., 2016). In addition, RAS-induced macropinocytosis is regulated by the translocation of vacuolar ATPase (v-ATPase) from intracellular membranes to the plasma membrane (Ramirez et al., 2019).

TRPML1 is a downstream effector of PIKfyve which partially regulates vacuole size (Krishna et al., 2016). It is a lysosome Ca^2+^ channel regulated by PI(3,5)P2. Lysosomes can modify macropinosomes isolated from TRPML1 silencing cells, but they do not fuse with each other (Dayam et al., 2015). PIKfyve and PI(3,5)P2 activate TRPML1 to induce macropinosome–lysosome fusion. Melanoma cells lacking TRPML1 exhibit lower survival, proliferation, tumor development, and macropinocytosis (Kasitinon et al., 2019). The filamentous septin GTPases connect preferentially with mature macropinosomes and localize to their fusion sites with macropinosomes in a phosphatidylinositol 3,5-bisphosphate–dependent manner. Septin deficiency leads to large clusters of docked macropinosomes that persist for longer and undergo fewer fusion events (Dolat and Spiliotis, 2016). This indicates that PIKfyve, TRPML1, and septin are involved in macropinosome degradation.

## Macropinocytosis and Tumor Cells

### Benefits of Macropinocytosis for Cancer Cells

#### Macropinocytosis Promotes Cell Growth and Survival

**FIGURE 2:**
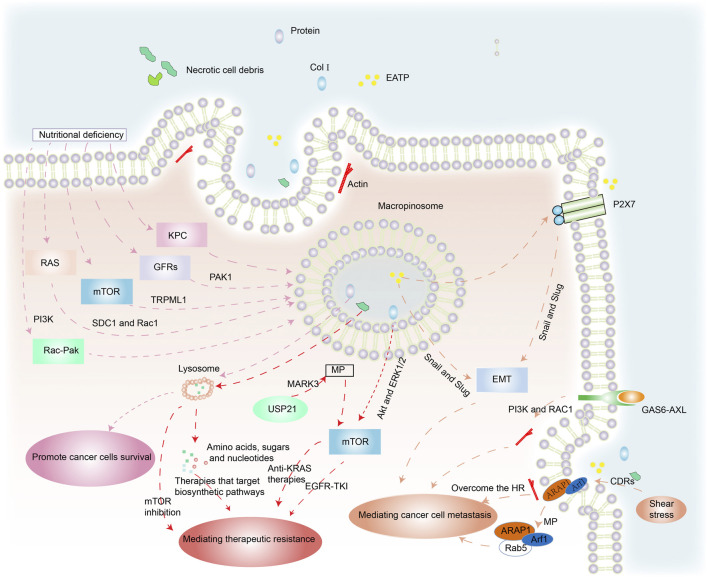
The benefits of macropinocytosis for cancer cells. (1) Under the pressures of nutrient deficiency, regulation of RAS genes, mTOR, GFRs and other factors triggers macropinocytosis in cancer cells. Macropinosomes fuse with lysosomes and foreign protein is degraded to amino acids that are released to enhance cancer survival. (2) Necrotic cell debris ingested by cancer cells acts against therapies that target biosynthetic pathways. USP21 promotes KRAS-independent tumor growth *via* macropinocytosis induced by MARK3, contrary to anti-KRAS therapies. Col I is also internalized by macropinocytosis, which mediates the resistance of EGFR-TK1 by mTOR activation through Akt and ERK1/2 independent pathways. (3) EATP induces EMT *via* enhanced transcription factors Snail and Slug (and possibly others) and directly and indirectly accelerates cell migration and invasion. GAS6-AXL signaling induces PI3K- and Rac1-dependent, actin-driven cytoskeletal rearrangements and macropinocytosis that jointly promote cancer migration. The position of cells insensitive to HR mediated by macropinocytosis enhances their ability to metastasize. The stimulation of shear stress promotes ARAP1/Arf1 trafficking to support cancer cell migration.

Cancer cells can acquire extracellular nutrients from nutrient-poor environments *via* macropinocytosis (Figure 2). This process depends on the activation of the RAS gene, growth factor receptors (GFRs), and other signal pathways (Hesketh et al., 2020; Hobbs et al., 2020). KRAS-transformed pancreatic cancer cells activate macropinocytosis to absorb and degrade extracellular fluids in order to survive (Hou et al., 2021). Membrane localization of trafficking syndecan-1 (SDC1) has been shown to be an essential mechanism for promoting macropinocytosis and sustaining the uncontrolled growth of oncogenic KRAS-driven pancreatic ductal adenocarcinoma (PDAC). SDC1 generates a signaling complex that activates Rac1 GTPase, which in turn stimulates PAK1 and activates macropinocytosis (Yao et al., 2019). Nonsmall cell lung cancer (NSCLC) exploits Rac-driven macropinocytosis of extracellular protein as an adaptive mechanism to survive glucose deprivation. Macropinocytosis is enhanced in these cells and is regulated *via* Rac-Pak signaling activation by phosphoinositide 3-kinase (PI3K) (Hodakoski et al., 2019).

Epidermal growth factor receptor (EGFR) enhances macropinocytosis during glutamine starvation by activating the serine–threonine kinase PAK1, which directly orchestrates the required alterations in membrane and cytoskeletal dynamics (Weerasekara et al., 2019). Lee et al. demonstrated that regional amino acid deficiency was associated with increased levels of macropinocytosis. This was mediated by nutritional stress-induced potentiation of EGFR signaling, which controlled the extent of macropinocytosis in PDAC *via* PAK1 activation (Lee et al., 2019).

The mammalian target of rapamycin (mTOR), a cellular pathway coordinating nutrient sensing and adaptive responses, has been implicated in macropinocytosis (Cang et al., 2013). mTOR inhibition elevated lysosomal catabolism of extracellular proteins and promoted cancer cell survival under nutrient-depleted conditions (Palm et al., 2015). In melanoma cells, TRPML1 is essential to inhibit the MAPK pathway and mTORC1 signaling. Macropinocytosis is reduced in TRPML1-deficient melanoma cells but is partially or totally restored by mTORC1 inhibition. TRPML1 expression is increased in melanoma cells compared to melanocytes, which suppresses MAPK and mTORC1 signaling, maintains macropinocytosis, and prevents proteotoxic stress (Kasitinon et al., 2019).

Xiao et al. demonstrated that Rab GTPases significantly increased in chRCC specimens and induced membrane trafficking and vesicle formation. Furthermore, the phospholipase C gamma 2 (PLCG2)/inositol 1,4,5-trisphosphate (IP3)/Ca2þ/protein kinase C (PKC) pathway regulated the activation of macropinocytosis and promoted chRCC cell proliferation under nutrient stress (Xiao et al., 2020). Thus, the oncogenic KRAS gene, GFRs, mTORC1, and other factors are highly important for macropinocytosis-mediated cancer cell survival in nutrient-deficient environments. These studies inform the development of effective anticancer agents.

#### Macropinocytosis and Therapeutic Resistance

Therapeutic resistance is a significant challenge to effective cancer treatment, especially in patients with aggressive cancers in which mutations that drive macropinocytosis are widespread. Hypoxia and nutrient deprivation are common in rapidly growing malignant tumors due to limited blood supply. This results in necrotic cell death in the core of solid tumors (Lee et al., 2018). The cancer cells ingest the necrotic cell debris *via* macropinocytosis, utilizing the amino acids, fatty acids, sugars, and nucleotides for biosynthesis, which makes them resistant to therapies that target anabolic pathways (Jayashankar and Edinger, 2020).

Collagen type I (Col I) induces EGFR tyrosine kinase inhibitor (EGFR-TKI) resistance by mTOR activation *via* Akt and ERK1/2 independent pathways (Yamazaki et al., 2018). 5-(N-ethyl-N-isopropyl) amiloride (EIPA) is a macropinocytosis inhibitor. EGFR-TKI resistance developed in EGFR-mutated lung cancer cells by Col I absorption *via* macropinocytosis, while macropinocytosis inhibition reduced Col I absorption in PC-9 cells and restored their EGFR-TKI sensitivity (Yamazaki et al., 2020).

The regulation of microtubule affinity-regulating kinase 3 (MARK3), a kinase regulator of cytoskeleton dynamics, is involved in macropinocytosis. Changes in KRAS (KRAS^∗^) are found in virtually all PDAC cases and are linked to tumor survival. Hou et al. identified a deubiquitinase USP21-driven mechanism that was resistant to KRAS^∗^ treatment. USP21 promotes KRAS^∗^-independent cancer development *via* the mTOR signaling pathway by MARK3-mediated up-regulation of macropinocytosis, which helps to fulfil the anabolic requirements (Hou et al., 2021).

Inhibition of autophagy can limit the progression of existing tumors and increase responsiveness to cancer treatment (Amaravadi et al., 2019). However, when PDAC is treated with autophagy inhibitors, the cancer cells take up exogenous proteins *via* macropinocytosis induced by NRF2, which provides the metabolites and energy required to develop therapeutic resistance (Su et al., 2021).

A recent study found that macropinocytosis reduces sensitivity to mTOR inhibition by restoring AKT phosphorylation at serine 473, and leading to cancer cell proliferation. This macropinocytosis-mediated resistance was eliminated when mTOR and lysosomal degradation of absorbed protein were inhibited (Michalopoulou et al., 2020). Thus, macropinocytosis allows cancer cells to develop resistance to antitumor drug therapies, making them ineffective. However, macropinocytosis inhibition is a potential therapy to combat this resistance.

#### Macropinocytosis and Metastasis

Systemic metastasis of cancers is the main prelude to mortality in cancer patients. The detection of metastasis using clinical imaging methods usually indicates a poor prognosis (Klein, 2020; Qin et al., 2020). Metastasis is a major challenge because of its complexity. It is associated with the development of epithelial–mesenchymal transition (EMT) in a wide range of cancers (Jiao et al., 2020; Fuh et al., 2021). Extracellular ATP (eATP) can trigger EMT in cancer cells *via* purinoceptor signals activated by P2X7. It can also activate production of matrix metallopeptidases (MMPs) and promote lung cancer cell shedding, EMT, migration, and invasion. This induction happens outside the cell but the functions are internalized into the cell by macropinocytosis. During this process, the enhancing transcription factors Snail and Slug (and probably others) may promote EMT (Cao et al., 2019).

The TAM receptor AXL and its ligand, GAS6, have been linked to the development and metastasis of various malignancies (Falcone et al., 2020). Zdalik-Bielecka et al. demonstrated that GAS6-AXL signaling induced PI3K- and Rac1-dependent, actin-driven cytoskeletal rearrangements and macropinocytosis that jointly promoted cancer invasion and metastasis (Zdzalik-Bielecka et al., 2021).

Actomyosin is recruited to the cell front of immature dendritic cells to produce macropinosomes. As a result, these cells are unaffected by hydraulic resistance (HR) because macropinocytosis allows fluid to pass through them. This mechanism enhances their ability to expand into other body spaces and may also enhance the ability of cancer cells to invade and metastasize (Moreau et al., 2019).

Circular dorsal ruffles (CDRs) are ring-shaped membrane structures that are rich in F-actin. Shear pressure promotes CDR development through the integrin-connected kinase (ILK)-ADP-ribosylation factors (ARAP1/Arf1) cascade. Shear stress can induce the establishment of intracellular macropinocytosis after CDR depolymerization, promoting ARAP1/Arf1 trafficking from macropinocytosis to early endosome marker Rab5 to facilitate cancer cell migration (Qin et al., 2021).

Yao et al. showed that aborting macropinocytosis by deletion of the HSPG receptor SDC1 might reduce lung metastatic potential and make pancreatic cancer cells less clonogenic (Yao et al., 2019). Choi et al. found that the number of metastatic nodules was dramatically reduced when RAS3 cells pretreated with EIPA were delivered intravenously into immune-deficient mice (Choi et al., 2021). Inhibiting macropinocytosis function is likely to significantly slow down metastasis and reduce cancer-related deaths.

### Negative Impacts of Dysregulated Macropinocytosis on Cancer Cells

**FIGURE 3:**
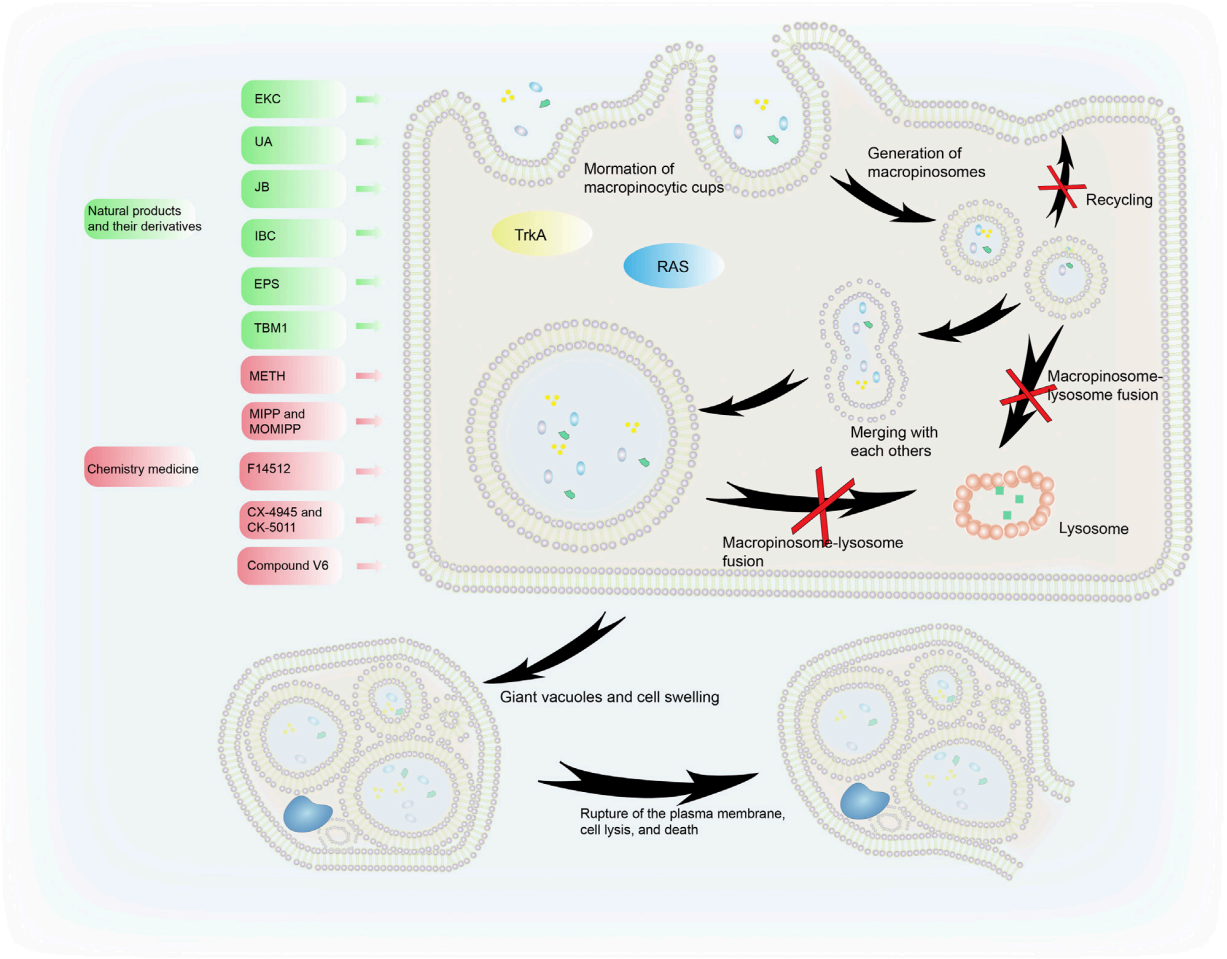
The process of methuosis. Several compounds induce methuosis. Over-activated RAS gene and TrkA can induce methuosis. Macropinosomes gradually merge to form large vacuoles which eventually leads to cell death. Natural products and their derivatives (such as EKC, UA, IBC, and JB) can induce methuosis, as can synthetic compounds such as METH, F14512, CX-4945, and CK-5011.

Cancer cells sustain their vitality through macropinocytosis when the nutrient and growth factors in the tumor microenvironment (TME) change. However, hyperstimulation of macropinocytosis by active RAS expression or some medicines can cause cell death, also known as methuosis. Methuosis occurs when cells accumulate too much fluid through uncontrolled macropinocytosis (Figure 3). In the normal macropinocytosis process, folds in the plasma membrane (PM) produce macropinocytic cups that close to form macropinosomes inside the cell. Circulating receptors transport some macropinosomes back to the PM (Toh et al., 2019), while other macropinosomes fuse with lysosomes and are cleaved (Maltese and Overmeyer, 2015).

Liquid uptake *via* macropinocytosis is enhanced during methuosis. Instead of shrinking, the vesicles swell, do not acidify, remain nonfunctional, and merge with each other rather than with lysosomes. This creates large vacuoles and catastrophic cell swelling that result in bursting of the PM, cell lysis, and death (Ritter et al., 2021). When activated Ras enters cancer cells, it causes the formation of large phase-lucent cytoplasmic vacuoles, leading to methuosis (Dendo et al., 2018). Endosome morphological changes associated with Ras-induced methuosis are caused by Rac1 downstream activation and Arf6 reciprocal inactivation (Bhanot et al., 2010). Activating the receptor tyrosine kinase TrkA overstimulates macropinocytosis in non-Ras altered medulloblastoma brain tumors, leading to catastrophic membrane disintegration and methuosis (Li et al., 2010). The molecular basis of this uncontrolled TrkA-driven macropinocytosis is the inhibition of RhoB that relieves actin stress fibers, and FRS2-scaf folded Src and H-Ras activation of RhoA that stimulates actin reorganization and the formation of lamellipodia (Li et al., 2016).

## Macropinocytosis and the Tumor Microenvironment

### Macropinocytosis and Cancer-Associated Fibroblasts (CAFs)

**FIGURE 4:**
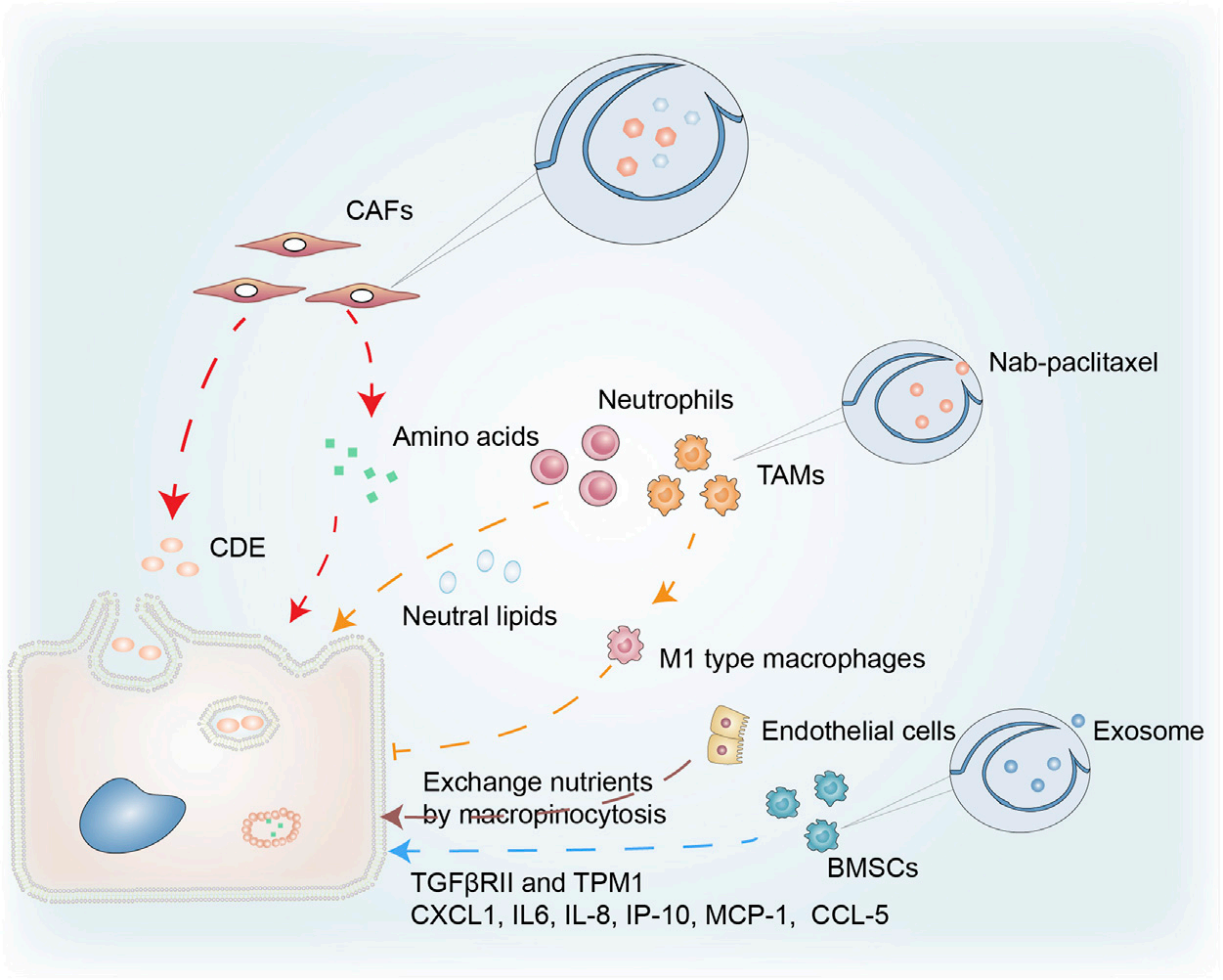
Macropinocytosis in the tumor microenvironment. Macropinocytosis in CAFs provides PDAC with a source of amino acids, while CDEs taken up by cancer cells through pathways similar to macropinocytosis promote tumor growth. Neutral lipids accumulating in lung neutrophils are transferred to metastatic tumor cells *via* the macropinocytosis-lysosome pathway, enhancing their survival and proliferation. Nab-paclitaxel absorbed *via* macropinocytosis enhances the polarization of M1-type macrophages and the inhibition of tumor cells. Endothelial cells in PCs have characteristics that facilitate macropinocytosis that promotes cancer growth. Macropinocytosis by BMSCs is important in cancer progression *via* uptake of exosomes.

CAFs are a prominent cellular component of the TME in most solid tumors and macropinocytosis in CAFs correlates with cancer growth (Figure 4). Zhang et al. demonstrated that glutamine deprivation triggered macropinocytosis in pancreatic CAFs. They discovered that stromal macropinocytosis was aided by increased cytosolic Ca^2+^ and was dependent on ARHGEF2 and CaMKK2-AMPK signaling. This macropinocytosis had two purposes: as a source of intracellular amino acids to maintain the functionality of the CAF cells, and to feed the PDAC cells with secreted amino acids that helped them avoid the negative effects of nutritional stress in the TME (Zhang Y. et al., 2021).

The Ras inhibitor, RASAL3 (epigenetically silenced in human prostatic CAFs) results in oncogenic Ras activity that promotes macropinocytosis-mediated glutamine synthesis. In prostate cancer, stromal glutamine acts as a source of energy *via* anaplerosis and as a facilitator of neuroendocrine differentiation. The suppression of macropinocytosis and glutamine transport has anticancer effects in an orthotopic xenograft model (Mishra et al., 2018). In addition, Zhao et al. found that CAF-derived exosomes (CDEs) increased glycolysis and glutamine-dependent reductive carboxylation in cancer cells by inhibiting mitochondrial oxidative phosphorylation. Using ^13^C-isotope labeling, they showed that exosomes delivered amino acids to cancer cells in a manner similar to macropinocytosis (Zhao et al., 2016).

### Macropinocytosis and the Cancer Immune Response

Dendritic cells trigger an immune response by presenting a polypeptide-MHC complex on their surface. T cells recognize and are activated by this complex. The ability of dendritic cells to efficiently present such complexes is partly due to their ability to capture antigens through macropinocytosis (Liu and Roche, 2015). The normal physiological functioning of macrophages, B cells, and T cells is inseparable from macropinocytosis (Redka et al., 2018; Lin et al., 2020). Under amino acid (AA) replete conditions, macropinocytosis enables extracellular AA to enter an endolysosomal compartment in T cells, allowing mTORC1 to remain activated and stimulating T cell proliferation (Charpentier et al., 2020). The relationship between macropinocytosis and immune cells in the TME is illustrated in Figure 4. Neutral lipids accumulating in lung neutrophils were transferred to metastatic tumor cells *via* a macropinocytosis-lysosome pathway. This enhanced the survival and proliferation of the tumor cells, while pharmacological suppression of macropinocytosis significantly reduced metastatic colonization by breast tumor cells (Li et al., 2020).

Treatment resistance in PDAC is partly attributable to responses including the general infiltration of immunosuppressive macrophages (M2), whilst type M1 macrophages inhibit tumor growth (Avila-Ponce de Leon et al., 2021). Macrophages ingest nanoparticle albumin-bound (nab)-paclitaxel primarily through macropinocytosis, which is sufficient to cause macrophage M1 polarization. This nab-paclitaxel-mediated M1 activation may result in positive feedback signaling, increasing drug uptake and strengthening M1-activating effects in both an autocrine and paracrine manner (Cullis et al., 2017). Further investigation is required into the role of macropinocytosis in immune cells in cancer.

### Macropinocytosis and Other Cell Types in Cancer

Macropinocytosis in other cell types has been connected to cancer survival in the TME (Figure 4). Bone marrow-derived mesenchymal stem cells (BMSCs), a component of the TME, play an essential role in cancer (Zhang et al., 2019). Transforming growth factor β receptor II (TGFβRII), with its complex functions in cancer progression, is often mutated in cancers. Exosomes produced by PC12 cells can enter BMSCs *via* macropinocytosis and transfer microRNAs into these cells. miR-21 can reduce the expression of TGFRII and tropomyosin-1 (TPM1) in BMSCs. As a result, tumor exosomes may regulate cancer progression by lowering TGFRII and TPM1 levels in normal cells (Tian et al., 2014). The communication between multiple myeloma (MM) cells and BMSCs is pivotal in MM cell development, proliferation, and treatment resistance. Macropinocytosis was partially involved in the uptake of exosomes from MM cells, while exosomes containing miR-146a could be transported into BMSCs. The secretion of various cytokines and chemokines (including CXCL1, IL6, IL-8, IP-10, MCP-1, and CCL-5) increased after miR-146a was overexpressed in BMSCs, leading to increased MM cell survival and migration (De Veirman et al., 2016; Zheng et al., 2019).

Ma et al. analyzed transmission electron micrographs of endothelial cells in pancreatic cancer (PC) and found several extended projections on the luminal surface that resembled macropinocytic filopodia, suggesting the presence of characteristics conducive to macropinocytosis. PC endothelial cells may be the primary location in the microvasculature net where nutritional exchange occurs, and macropinocytosis may facilitate the exchange of nutrients or waste that promotes cancer growth (Ma et al., 2020).

## Anticancer Therapies Targeting Macropinocytosis

### Exploitation of Macropinocytosis in Cancer Treatment

#### The Use of Macropinocytosis-Inducing Carriers

Despite breakthroughs in cancer therapies and the development of personalized medicine, chemoresistance remains a significant factor contributing to cancer mortality (Chern and Tai, 2020). However, macropinocytosis can increase the transport of anticancer agents into cancer cell lines, rendering chemotherapeutic drugs more effective against drug-resistant cells (Muley et al., 2020). Studies have focused on combining substances that induce macropinocytosis with anticancer agents that are not dependent on macropinocytosis. The efficacy of anticancer drugs is improved by the formation of such conjugates. Extracellular vesicles (EVs) (including exosomes) play a crucial role in cell information communication and have potential as drug carriers (Costa Verdera et al., 2017). Nakase et al. described a technique for inducing active macropinocytosis to increase cellular EV uptake using arginine-rich, cell-penetrating peptide (CPP)-modified EVs. The macropinocytosis pathway was activated after the arginine-rich CPPs on the EV membrane were modified, with the number of arginine residues affecting cellular EV absorption efficiency. Subsequently, anticancer activity was achieved using ribosome-inactivating, protein saporin-encapsulated EVs modified with hexadeca-arginine (R16) peptide (Nakase et al., 2017).

Exosomes produced by normal fibroblast-like mesenchymal cells are designed to contain siRNA or shRNA specific to oncogenic KRAS^G12D^ (iExosomes), a frequent pancreatic cancer mutation. iExosomes exhibit higher efficacy than liposomes for targeting oncogenic KRAS. This is dependent on CD47 and aided by macropinocytosis. Multiple pancreatic cancer mice models have been treated with iExosomes, inhibiting tumor growth and improving survival rates (Kamerkar et al., 2017).

Neutral red (NR) is known for its ability to enter cells by a process similar to macropinocytosis, while histone deacetylase (HDAC) plays a vital role in the transcriptional regulation of tumor suppressor genes and oncogenes (Cui et al., 2021). The anticancer effects of HDAC inhibitors such as trans-cinnamic acid (TCA) are known (Kim and Bae, 2011; Zhu et al., 2016). Zhu et al. demonstrated that cinnamoyl phenazine composed of NR and TCA entered cancer cells *via* macropinocytosis and exhibited anticancer effects in pancreatic carcinoma xenografts (Zhu et al., 2017).

Albumin accumulates in certain tumor cells *via* macropinocytosis, so albumin-coupled cytotoxic drugs can be designed to carry anticancer agents (Lagiewka et al., 2021). Nab-paclitaxel, a widely applied clinical nanomedicine, is a nano-albumin-bound drug that leverages the therapeutic potential of macropinocytosis (Adkins et al., 2021).

#### The Use of Macropinocytosis-Inducing Drugs

**FIGURE 5:**
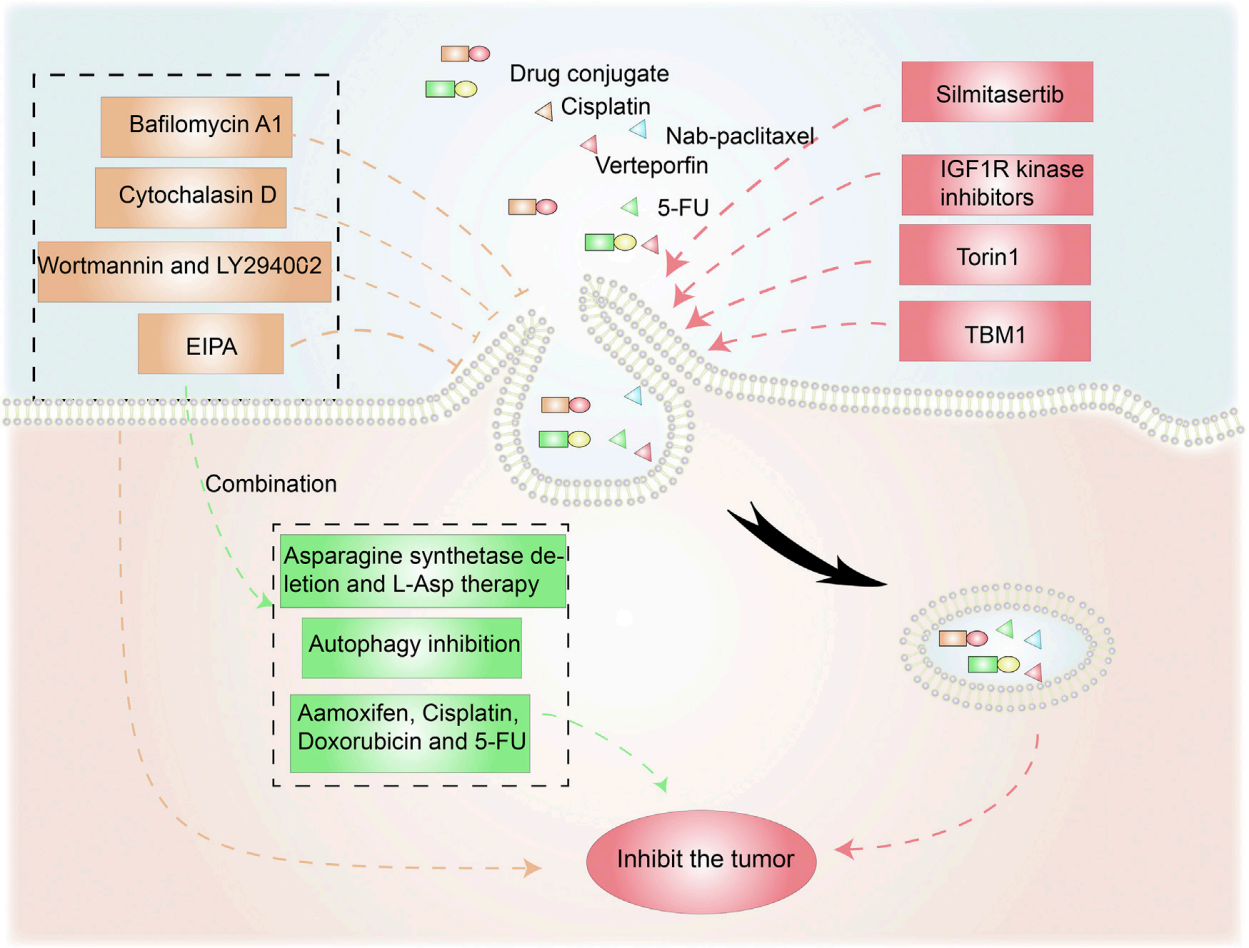
Anticancer therapies targeting macropinocytosis. Drug conjugates containing compounds that activate macropinocytosis in addition to anticancer drugs promote cancer treatment. Moreover, macropinocytosis-initiating drugs combined with nab-paclitaxel or verteporfin inhibit tumor cells. Macropinocytosis inhibitors such as cytochalasin D, Wortmannin/LY290042, and EIPA can affect tumor survival and drug resistance. EIPA combined with asparagine depletion and tamoxifen *etc*. can have synergistic antitumor effects.

In these previous studies the active medication was coated with, or coupled to, a molecule that caused tumor cells to undergo macropinocytosis. However, a more straightforward option is to use a macropinocytosis inducer to directly increase macropinocytotic extracellular drug absorption by cancer cells (Figure 5). The IGF1R kinase inhibitor AXL1717 enhanced the uptake and efficacy of nab-paclitaxel by mimicking glucose deprivation and promoting macropinocytosis *via* APMK, a cellular nutrient sensor (Li et al., 2021). KRAS-enhanced macropinocytosis and reduced FcRn-mediated recycling make pancreatic cancer cells sensitive to albumin-conjugated drugs, such that the drugs are more likely to accumulate in cancer cells (Liu et al., 2019).

Torin1-mediated mTORC1/2 inhibition increases macropinocytosis, whereas verteporfin-induced YAP inhibition causes apoptosis. Torin1-induced macropinocytosis promoted the absorption of verteporfin, boosting its pro-apoptotic properties in breast cancer cells (Dai et al., 2021). Song et al. demonstrated that silmitasertib (formerly CX-4945) caused macropinocytosis in oral squamous cell carcinoma, which could boost cisplatin intracellular uptake and promote apoptosis (Song et al., 2021).

Tubeimoside-1 (TBM1) is a low-toxicity triterpenoid saponin derived from *Bolbostemma paniculatum*, a traditional Chinese herb. It efficiently trafficked dextrans into heterotopic xenografts *via* macropinocytosis. High performance liquid chromatography analysis showed that 5-fluorouracil (5-FU) content dramatically increased in tumor tissues pretreated with TMB1 (Gong et al., 2018). Drugs that induce macropinocytosis when combined with anticancer medications eliminate the need for time-consuming nanoparticle or conjugate syntheses.

### Disrupting Macropinocytosis Suppresses Cancer Cells

#### Inhibitors of Macropinocytosis

Given the benefits of macropinocytosis for tumor growth, inhibition of macropinocytosis can impact tumor survival and drug resistance (Figure 5) (Hussein et al., 2021). Cytochalasin D, an inhibitor of actin polymerization, suppresses membrane ruffling and macropinocytosis induction in fibroblast cells (Stockinger et al., 2006; Sipos et al., 2019). The PI3K inhibitors wortmannin and LY294002 inhibit the closure of macropinosomes in fibroblasts and macrophages by suppressing the PI3K signaling pathway (Amyere et al., 2000; Araki et al., 2007). The sodium/hydrogen exchange inhibitors amiloride, HOE-694, and EIPA negate EGF-induced macropinocytosis by decreasing the sub-membranous pH (Koivusalo et al., 2010). Bafilomycin A1, a v-ATPase inhibitor that affects lysosomal pH, also prevents macropinocytosis (Kitazawa et al., 2017).

#### Synergistic Antitumor Effects of Macropinocytosis Inhibition

As an energy access pathway, macropinocytosis can influence proliferation, metastasis, and metabolic alterations in cancer. However, it is only one cancerous metabolic pathway and there are other survival strategies under nutrient-poor conditions. Consequently, synergistic antitumor effects are possible when macropinocytosis inhibition is combined with anticancer drugs (Figure 5). L-asparaginase, a critical component of acute lymphoblastic leukemia therapy, catabolizes L-asparagine to L-aspartate, causing extracellular asparagine to be depleted (Chan et al., 2014). Hanada et al. reported that asparagine depletion (following a combination of asparagine synthetase deletion and L-asparaginase therapy) combined with EIPA treatment suppressed tumor growth in KRAS-mutant CRC cells (Hanada et al., 2021).

Su et al. discovered that the ULK1/2 inhibitor MRT68921 and EIPA on their own had only minor effects on KC6141, 1444 and 1334 tumor growth. However, when combined, the two medicines had a significant influence on growth and lowered intracellular ATP and amino acids (Su et al., 2021). In poorly perfused locations where nutrients are scarce, necrotic cell debris ingested *via* macropinocytosis can support tumor cell anabolism (Kim et al., 2018). In the presence of standard-of-care chemotherapies that target biosynthetic pathways, the nutrients obtained from necrocytosis may allow cells to proliferate. Therefore, when used in conjunction with regular chemotherapy, macropinocytosis inhibitors have the potential to improve survival rates in patients with potentially fatal or aggressive malignancies (Puccini et al., 2022). A recent study found that EIPA combined with tamoxifen had a synergistic cytotoxic effect in MCF-7 breast cancer cells. EIPA also enhanced the curative effects of cisplatin, doxorubicin, and 5-FU on breast cancer, but the relationship with macropinocytosis inhibition needs further research (Rolver et al., 2020). Unlike the induction of macropinocytosis, which increases antineoplastic drug absorption, macropinocytosis inhibitors can offer antitumor therapies that disrupt the metabolic activity of cancer cells (Table 1).

**TABLE 1:** Examples of use of macropinocytosis in cancer therapy.

Drug	Effect on macropinocytosis	Macropinocytosis target medicine	Type of cancer	Mechanism	Reference
Torin1	Promotion	Verteporfin	Breast cancer	Torin1-induced macropinocytosis led to increased verteporfin intake	Dai et al. (2021)
Silmitasertib	Promotion	Cisplatin	Oral squamous cell carcinoma	Silmitasertib-induced macropinocytosis led to increased cisplatin intake	Yang et al. (2019)
Tubeimoside-1	Promotion	5-FU	Colorectal cancer	Tubeimoside-1-induced macropinocytosis led to increased 5-FU intake	Gong et al. (2018)
Cytochalasin D	Inhibition	None	RAS-driven cancer	Inhibition of actin polymerization	Sipos et al. (2019), Stockinger et al. (2006)
Wortmannin and LY294002	Inhibition	None	RAS-driven cancer	Inhibition of PI3K signaling pathway	Amyere et al. (2000), Araki et al. (2007)
EIPA	Inhibition	MRT68921	Pancreatic ductal adenocarcinoma *etc*.	Tumor starvation	Su et al. (2021)
Bafilomycin A1	Inhibition	None	RAS-driven cancer	Altered lysosomal pH	Kitazawa et al. (2017)

#### Methuosis-Inducing Compounds

Although various anticancer agents are clinically available, the occurrence of intrinsic or acquired resistance is a major obstacle to the use of chemotherapy drugs and targeted therapies. Drug resistance in cancer cells is intimately linked to various mechanisms including increased rates of drug efflux, changes in drug metabolism, and drug target mutations (Holohan et al., 2013; Wang et al., 2020). Many chemotherapeutic agents function by triggering intrinsic apoptosis, so it is reasonable to seek alternative cell death pathways (such as methuosis) to target apoptosis-resistant tumor cells. Various natural products or their derivatives, in addition to synthetic drugs, can induce methuosis (Figure 3). These provide a basis for further clinical treatments.

##### Natural Products and Derivatives

Many medications derived from natural products or their derivatives have been recognized as potential phytochemical cancer therapies that utilize methuosis (Table 2). Induction of methuosis in cancer cells is one of the advantages of natural product antitumor therapies, and these research studies are aiding the development of new anticancer strategies.

**TABLE 2:** Methuosis-inducing compounds.

Drug	Origin	Type of cancer	Mechanism	Reference
EKC	Pentenylated flavonoid isolated from the Chinese herbal medicine epimedium	NCI-H292 and A549 lung cancer cells	Rac1 and Arf6-regulated macropinosomes fused, forming large vacuoles resulting in methuosis	Liu et al. (2021)
IBC	Naturally occurring chalcone compound	Leukemia cells	Vacuolar-type H^+^-ATPase and AKT involved in methuosis-like death	Yang et al. (2019)
UA	Pentacyclic triterpenoid carboxylic acid	HeLa cells *etc*.	Excessive vacuoles	Sun et al. (2017)
PD	Extracted from the root of *Platycodon grandiflorum*	A549 and MCF7 cells	Excessive vacuoles	Jeon et al. (2019)
JB	Natural sphingolipid	HGC-27 cells	Excessive vacuoles	Cingolani et al. (2017)
EPS	The infructescence of *Platycarya Strobilacea Sieb. et Zucc.*	Nasopharyngeal carcinoma cells	Transcription factor c-FOS and related genes may regulate methuosis	Liu et al. (2020)
Spiropachysine A	Extracted from *Pachysandra axillaries Franch.* var. styiosa (Dunn) M. Cheng	MHCC-97H cells	Ras/Rac1 signaling partially involved in methuosis	Fang et al. (2022)
TBM1	Triterpenoid saponin derived from *Bolbostemma paniculatum*	SW480 cells	Excessive vacuoles	Gong et al. (2018)
METH	Psychostimulant drug	Neuroblastoma cells	Activation of Rac/Rac1 induced defective lysosomal function	Nara et al. (2012)
MIPP and MOMIPP	Indole-based chalcones	U251 glioblastoma cells	Disruption of Rab5 GTPase	Robinson et al. (2012)
F14512	Polyamine-modified topoisomerase II inhibitor	A549 cells	Excessive vacuoles	Brel et al. (2011)
Compound 13	4,6-disubstituted azaindole	MDA-MB-231 *etc*.	Excessive vacuoles	Huang et al. (2018)
Vacquinol-1	Quinine derivative	Glioblastoma cells	Excessive vacuoles	Ahlstedt et al. (2018)
CX-4945 and CX-5011	Protein kinase CK2 inhibitors	HepG2 cells	Uncontrolled Rac1 activation promoted vesicle accumulation	D'Amore et al. (2020)
Compound V6	S-triazine compound	U87 glioblastoma cells	Formation of H-bonds with ^273^R and ^276^Y in vimentin rod domain	Zhang L. et al. (2021)

Epimedokoreanin C (EKC) is a pentenylated flavonoid extracted from the Chinese herbal medicine epimedium. Liu et al. describe how EKC reduces cell viability and causes significant vacuolation in human lung cancer cells. Rac1 and Arf6 regulation was partially involved in this methuosis. Furthermore, the effects of doxorubicin and etoposide were enhanced by EKC, suggesting that EKC may be a beneficial complement to conventional chemotherapy (Liu et al., 2021). Isobavachalcone (IBC), a naturally occurring chalcone, is extracted from traditional Chinese medicines such as *Psoralea corylifolia L.* (Kuete and Sandjo, 2012). It selectively causes non-apoptotic, methuosis-like cell death in leukemia cells. Two unique features of methuosis are the activation of vacuolar-type H^+^-ATPase and AKT (Yang et al., 2019).

Ursolic acid (UA), a pentacyclic triterpenoid carboxylic acid extracted from Chinese herbal medicines, exhibits significant anticancer activity by triggering p53-mediated cell cycle arrest and apoptosis (Yin et al., 2018). Sun et al. described the discovery of a UA-derived small molecule (compound 17) that caused cancer cell death *via* hyperstimulation of macropinocytosis. Cell death halted when stimulation was inhibited by the macropinocytosis inhibitor amiloride, simultaneous with methuosis (Sun et al., 2017). Platycodin D (PD), extracted from the root of *Platycodon grandiflorum*, enhances human immunity and is one of the main saponins used against tumors (Zhao et al., 2015). Jeon et al. proposed that PD-induced cell death with extreme vacuolation constituted methuosis (Jeon et al., 2019).

Jaspine B (JB), a natural sphingolipid, is isolated from marine sponges (Canals et al., 2009). It caused atypical cell death of HGC-27 cells characterized by cytoplasmic vacuoles developing in a time- and dose-dependent manner. Vacuoles seemed to form as a result of macropinocytosis, causing cytoplasmic disruption. JB joins the list of substances that cause cytoplasmic vacuolation and methuosis (Cingolani et al., 2017). An ethanolic extract of the infructescence of *Platycarya strobilacea Sieb. et Zucc.* (EPS) demonstrated potent antinasopharyngeal cancer properties *in vitro*. The production of CNE1 and CNE2 cell fusion and vacuoles, the disruption of lysosomal vesicle transit, and the activation of methuosis were all linked to the anticancer effects of EPS (Liu et al., 2020).

Spiropachysine A is a traditional Chinese steroidal alkaloid extract that is extensively used to improve blood circulation and reduce pain and inflammation. Its antitumor activity is mediated through methuosis and is not dependent on classical cell death signaling pathways. Activation of the Rac1 signaling pathway appears to be required for methuosis (Fang et al., 2022). TBM1 causes the rapid production of a large number of vesicular-like structures. The primary mechanism of TBM1-induced vacuolization is macropinocytosis, which leads to methuosis.

Thus, it is evident that natural products and their derivatives have broad application in clinical antitumor therapies mediated *via* methuosis (Gong et al., 2018).

##### Synthetic Compounds

Non-natural compounds can also induce methuosis (Table 2). Methamphetamine (METH) primarily alters the integrity of dopamine terminal neurons and interferes with nerve conduction (Abbruscato and Trippier, 2018). When neuroblastoma cells were cultured in METH they lost their activity after macropinocytosis was activated by Ras and Rac1 (Nara et al., 2012).

Researchers reported a chalcone-like molecule, MIPP, that promoted methuosis in glioblastoma and other cancer cells. A derivative, MOMIPP, induced methuosis at low concentrations (Robinson et al., 2012). The JNK signaling pathway was involved in methuosis triggered by MOMIPP (Li et al., 2019). F14512, a polyamine-modified topoisomerase II inhibitor, is considered an effective anticancer drug candidate (Barret et al., 2008). Brel et al. showed that its anticancer effects were related to the accumulation of numerous vacuoles, as seen during methuosis (Brel et al., 2011).

The azaindole-based compound 13 caused MDA-MB-231 cells to vacuolate and die. It was cytotoxic towards a wide range of cancer cell lines, including MDA-MB-231, A375, HCT116, and MCF-7, with cell death being mediated by methuosis (Huang et al., 2018). Vacquinol-1, a quinolone derivative, induced glioblastoma multiforme cell death by methuosis and has promising therapeutic potential. Cell death was counter-regulated by exogenous ATP, which activated the transient receptor potential cation channel, subfamily M, member 7 (TRPM7) (Ahlstedt et al., 2018).

CK2, a pleiotropic enzyme deeply involved in cell proliferation and apoptosis, is considered a promising therapeutic agent against cancer (Manni et al., 2017; Fasolato et al., 2021). In cholangiocarcinoma and other cancer cell lines, CX-4945 treatment stimulated the formation of cytosolic vacuoles. Extracellular fluid at neutral pH was seen in the vacuoles, which is characteristic of methuosis (Lertsuwan et al., 2018). CX-5011, a CK2 inhibitor related to CX-4945, acts as a CK2-independent methuosis inducer with four times the potency of its parent compound and the ability to promote the creation of larger cytosolic vacuoles at low micromolar doses. The pharmacological suppression of small GTPase Rac1, siRNA-mediated downregulation of Rac1, and overexpression of the dominant-negative mutant form of Rac1 (Rac1 T17N) both reduced CX-5011’s ability to induce methuosis (D'Amore et al., 2020).

Vimentin is involved in the membrane trafficking and transporting of endolysosomal vesicles (Margiotta and Bucci, 2016). Zhang et al. created a variety of novel s-triazine compounds that caused methuosis in various cancer cells. For example, compound V6 exhibited striking methuotic properties and vimentin was its specific target protein. According to molecular docking analysis, V6 can form hydrogen bonds with vimentin at ^273^R and ^276^Y in its rod domain (Zhang L. et al., 2021). The molecular mechanisms involved in drug-induced methuosis remain unclear and require further study. Meanwhile, other methuosis inducers remain to be discovered.

## Conclusion and Perspectives

Tumor growth and drug resistance mediated by macropinocytosis have recently become hot topics in cancer tumor research, as illustrated by the many studies of the mechanisms, regulation, and applications of macropinocytosis. Understanding the mechanisms of macropinocytosis and its effects on tumor cells benefits the development of new therapeutic strategies. Studying drugs that promote macropinocytosis helps to improve the targeting and efficacy of macropinocytosis-dependent chemotherapeutic drugs. Anticancer medications can be combined with carriers or drugs that induce macropinocytosis. Meanwhile, inhibition of macropinocytosis cuts off an alternative pathway of cancer cell metabolism, reverses tumor resistance, and increases tumor sensitivity to anticancer drugs.

Methuosis inducers provide an alternative pathway to cancer cell death beyond programmed apoptosis and have powerful anticancer potential. Screening natural products for methuosis-inducing drugs is a viable strategy, but the mechanisms connecting cell death with extreme vacuolation require greater research.

Understanding the relationship between tumor cells and macropinocytosis in the TME is crucial to developing antitumor strategies. Macropinocytosis in the TME promotes tumor survival and blocking microenvironmental macropinocytosis can provide additional benefits to antitumor therapies. However, most studies are concerned with macropinocytosis in cancer cells and few address macropinocytosis related to the microenvironment. In addition, macropinocytosis inhibitors inhibit the growth of tumor cells but also impede the supportive effects that the TME provides to cancer cells. Cullis et al. confirmed that drugs affected microenvironmental components through macropinocytosis and provided strategies for tumor treatment (Cullis et al., 2017). The TME is extremely complex and the mechanisms by which macropinocytosis affects the survival of cancer cells need to be elucidated. The extent to which macropinocytosis in the TME affects tumor survival, metastasis, and therapy resistance remains unclear.

Challenges and problems remain in this area. For example, not all cancer cells exhibit the phenomenon of macropinocytosis, which may limit the application of treatment methods that target macropinocytosis. Means of identifying macropinocytosis in cells are also limited. Inhibitors (e.g., EIPA and cytochalasin D) have been routinely used to demonstrate macropinocytosis, while detecting the uptake of dextran, a macropinocytosis marker, is the most commonly used method. However, neither method specifically identifies macropinocytosis and researchers lack real-time, quantitative detection options in macropinocytosis research. Further research is required into techniques for effectively regulating macropinocytosis and related mechanisms. This review of the field highlights promising new methods and strategies for tumor treatment.
